# The effect of low volume high-intensity interval training on metabolic and cardiorespiratory outcomes in patients with type 2 diabetes mellitus: A systematic review and meta-analysis

**DOI:** 10.3389/fendo.2022.1098325

**Published:** 2023-01-04

**Authors:** Yang Peng, Yiran Ou, Ke Wang, Zhenghao Wang, Xiaofeng Zheng

**Affiliations:** ^1^ West China Hospital/West China School of Medicine, Sichuan University, Chengdu, China; ^2^ Department of Endocrinology and Metabolism, Center for Diabetes and Metabolism Research, West China Hospital, Sichuan University, Chengdu, China; ^3^ Department of Vascular Surgery, University Hospital of Chengdu University of Traditional Chinese Medicine, Chengdu, China; ^4^ The Rolf Luft Research Center for Diabetes and Endocrinology, Karolinska Institutet, Stockholm, Sweden

**Keywords:** low volume high-intensity interval training, meta-analysis, metabolism outcome, type 2 diabetes mellitus, cardiorespiratory outcomes

## Abstract

**Aims:**

The present systematic review and meta-analysis of randomized controlled trials (RCTs) was conducted to investigate the effect of low volume high-intensity interval training (LVHIIT) on the metabolic and cardiorespiratory outcomes in patients with type 2 diabetes mellitus (T2DM).

**Methods:**

Relevant articles were sourced from PubMed, EBSCO, Web of Science, Embase, and the Cochrane Library from inception to October 2022. The study search strategy and all other processes were implemented in accordance with the PRISMA statement.

**Results:**

Five randomized controlled trials that satisfied the inclusion criteria were included in this meta-analysis. The LVHIIT group had significantly lower fasting blood glucose levels (RR= -1.21; 95% CI= -2.02— -0.40, p = 0.0032) and HbA1c levels (RR= -0.65; 95% CI= -1.06— -0.23, p = 0.002) and higher levels of insulin resistance indicator HOMA-IR (RR= -1.34; 95% CI = -2.59— -0.10, p = 0.03) than the control group. Moreover, our results show that LVHIIT can reduce body mass (RR = -0.94, 95% CI = -1.37— -0.51, p<0.0001) and body mass index (RR = -0.31, 95% CI = -0.47— -0.16, p<0.0001). LVHIIT had a better therapeutic effect on blood lipid metabolism, such as total cholesterol, high-density lipoprotein, low-density lipoprotein and triglycerides. However, the change in fasting insulin levels was not statistically significant (RR= -1.43; 95% CI = -3.46— 0.60, p =0.17). Furthermore, LVHIIT reduced the systolic blood pressure (RR =-4.01, 95% CI = -4.82 – -3.21, p<0.0001) and improved peak oxygen uptake (VO_2peak_) compared to the control group (RR= 5.45; 95% CI = 1.38 – 9.52, p =0.009).

**Conclusion:**

After a certain period of LVHIIT, glycaemic control, insulin resistance, body weight, lipid profile and cardiorespiratory outcomes were significantly improved in T2DM patients.

## Introduction

Type 2 diabetes mellitus (T2DM) is a metabolic disease characterized by increased blood glucose concentrations. T2DM affects more than 400 million people worldwide, a figure that is expected to exceed 642 million people by 2040. In addition to severe suffering for the actual patient, the economic cost for disability and treatment places a heavy burden on society (1). Although many pharmacological treatments have emerged in recent years, these medications not only fail to prevent the progression of diabetes and its complications but also cause many side effects.

Cardiometabolic risk factors including central obesity, hypertension, dyslipidemia, and insulin resistance are strongly associated with the development and progression of T2DM (2). Lifestyle interventions appear as the efficient strategy to minimize cardiometabolic risk factors and improve T2D, which has gained increasing attention and acceptance among patients due to their simplicity and repeatability (3–5). Lifestyle interventions can be used as primary or supplementary treatments for T2DM patients according to the current ADA (American Diabetes Association) guidelines (6). Recent clinical trials have demonstrated that intensive lifestyle interventions can reduce the incidence of diabetes by 58% compared to those without lifestyle interventions (7). Among these lifestyle interventions, physical exercise results in improved insulin sensitivity and glucose homeostasis, which has long been recommended as one of the key therapeutic interventions for T2DM (8). High-intensity interval training (HIIT) consists of alternating repetitions of short periods of high-intensity exercise interspersed with less active or passive recovery periods. HIIT should be performed at 80–100% of the max heart rate interval, with a lower heart rate during the rest period. Compared to widely used moderate-intensity continuous training (MICT), HIIT has been proposed as a lower total energy expended exercise intervention that may bring about similar positive effects (9, 10). Collective evidence suggests that HIIT contributes to greater improvements in cardiorespiratory fitness compared to MICT (11), where cardiorespiratory fitness is inverse associated with the incidence of T2DM (12).

Nevertheless, normal HIIT has a much higher exercise intensity with a higher risk of injury in T2DM patients (13). Therefore, a milder HIIT exercise protocol is needed to reduce the risk of HIIT while improving metabolic and cardiorespiratory outcomes in individuals with T2DM. Additionally, lack of time is one of the common obstacles to physical activities. Low volume high-intensity interval training (LVHIIT) is a type of HIIT with the reduced total training volume (14). It has been suggested that LVHIIT could improve cardiorespiratory fitness as effective as high-volume HIIT, suggesting that LVHIIT may serve as a potent and time-efficient physical activity intervention strategy (15–17). The beneficial roles of LVHIIT on body composition have also been demonstrated (16, 18). However, the effects of LVHIIT on metabolic and cardiorespiratory outcomes in patients with T2DM remain unclear. In addition, new studies with more detailed data and high evidence levels have been published. Thus, we performed the current systematic review and meta-analysis of randomized controlled trials (RCTs) to investigate the effects of LVHIIT on T2DM patients. The results of this investigation may guide future decision-making regarding the use of lifestyle interventions among patients with T2DM.

## Materials and methods

This systematic review and meta-analysis followed the guidelines of the Preferred Reporting Items for Systematic Reviews and Meta-analysis (PRISMA) statement and the Cochrane Handbook for Systematic Reviews of Interventions (19). Ethical approval and patient consent were not required because all analyses were based on previously published studies.

### Literature search and selection criteria

LVHIT as intervention treatment vs no exercise or shame exercise (exercise at very low density). We systematically searched several databases including PubMed, EBSCO, Web of science, EMbase, and the Cochrane Library from inception to October 2022. The structured search strategies used the combination of RCTs and LVHIIT with diabetes patients: [“Low volume” OR “high-intensity interval training” AND (exercise OR physical activity) AND (“randomized controlled trials”) AND (diabetes) NOT (review) NOT (meta) NOT (animal experiment)]. The reference lists of retrieved studies and relevant reviews were hand-searched, and the process mentioned above was performed repeatedly to ensure the inclusion of all eligible studies. Inclusion criteria were as follows (1): randomized control trials, (2) T2DM diagnosis before participating in the experiment, (3)as there is no universal standard definition of HIIT, thus we used the HIIT standard proposed by previous meta-analysis (20, 21), (4) LVHIT as intervention treatment vs no exercise or shame exercise, (5) sufficient data for extraction, (6) full text only, and studies with all languages were included.

### Data extraction and outcome measures

Baseline information that was extracted from the original studies included the following: first author, published year, number of patients, patient age and gender distributions, the evaluation of the evidence level, detailed intervention method and time of period. Data were independently extracted by two investigators. Discrepancies were resolved by consensus.

The primary outcomes were fasting blood glucose (FBG), HOMA-IR and HbA1c. Secondary outcomes were fasting insulin, body mass index (BMI), body mass, plasma lipid metabolism (TC, HDL, LDL and triglyceride) and the cardiorespiratory fitness parameters including systolic blood pressure (SBP), diastolic blood pressure (DBP) and relative VO_2 peak_.

### Quality assessment of individual studies

The methodological quality of each RCT was assessed by the Jadad Scale which consists of three evaluation elements: randomization (0-2 points), blinding (0-2 points), dropouts and withdrawals (0-1 points) (22). One point was be allocated to each element if it had been conducted and mentioned appropriately in the original article. The total score of the Jadad Scale ranges from 0 to 5 points. An article with a total Jadad score that is less than or equal to 2 is considered to be of low quality. Concurrently, a study is thought to be of high quality if its total Jadad score greater or equal to 3 (23).

### Statistical analysis

Risk Ratio (RR) with 95% confidence intervals (CIs) was calculated for dichotomous outcomes. Heterogeneity was evaluated using the I^2^ statistic, with I^2^ > 50% taken to indicate significant heterogeneity (24). Sensitivity analysis was performed to evaluate the influence of a single study on the overall estimate by omitting one study in turn or performing subgroup analysis. The random-effects model was used for meta-analysis. Owing to the limited number of included studies (<10), publication bias was not assessed. Statistical significance was accepted at P < 0.05. All the data are presented as mean ± SD. All statistical analyses were performed using Review Manager Software Version 5.3 (The Cochrane Collaboration, Software Update, Oxford, UK).

## Results

### Literature searches, study characteristics, and quality assessment

**Figure 1:**
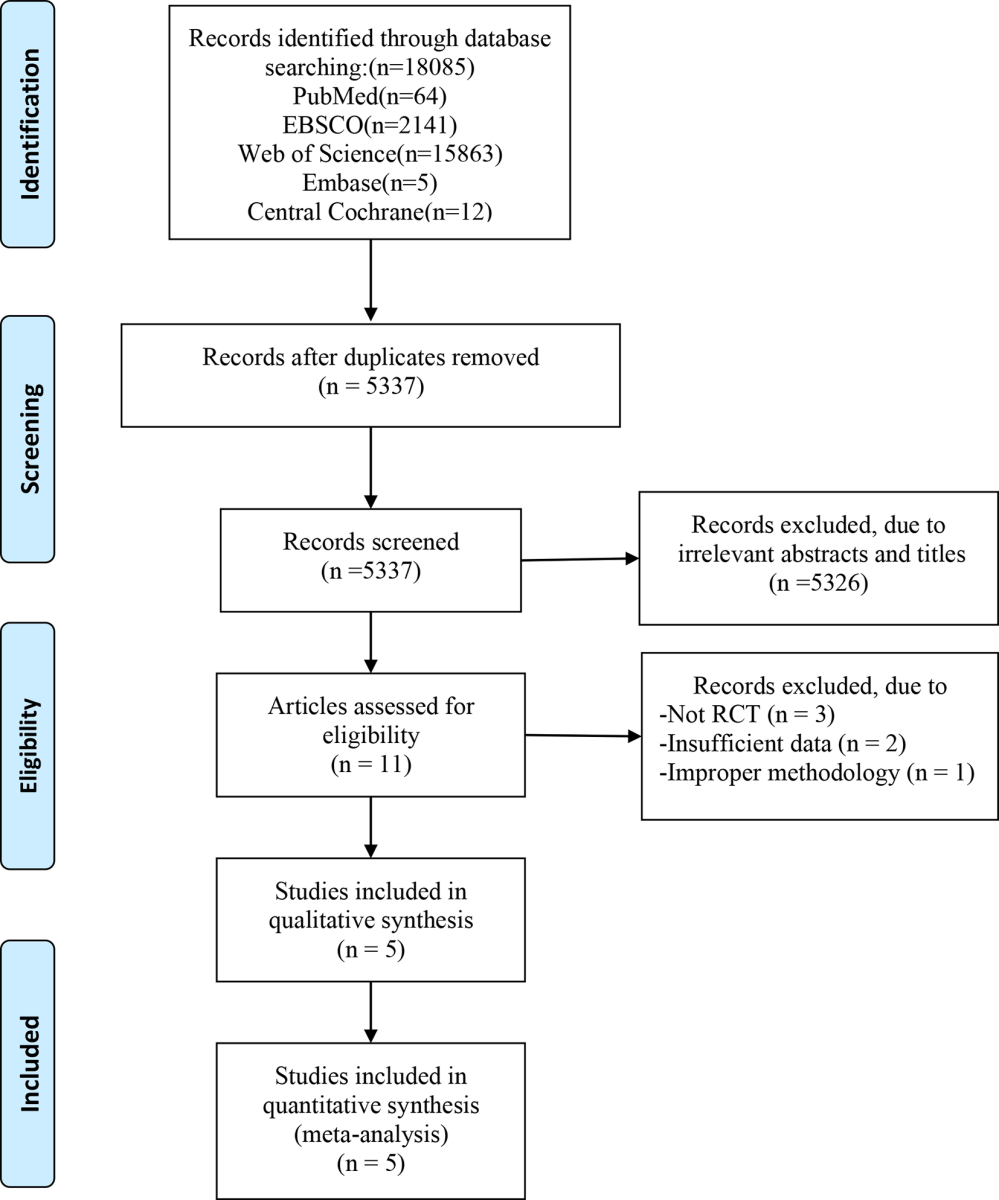
PRISMA flow chart.

In total, 18085 articles including 64 in PubMed, 2141 in EBSCO, 15863 in Web of science, 5 in Embase and 12 in central Cochrane were initially identified from the databases. After removing duplicates, 5537 articles were retained. A total of 5326 studies were excluded from our study due to unrelated abstracts and titles. We also excluded 3 studies that were not RCTs, 2 studies that presented insufficient data, and 1 study that reported an improper methodology. Ultimately, five RCTs satisfied the inclusion criteria and were included in this meta-analysis (10, 25–28). The article selection process was performed in accordance with the PRISMA statement and the flow chart is shown as **Figure 1**. The baseline characteristics of the 5 included studies are shown in **Table 1**. Only Afousi et al. reported the age range of the patients (45-60 years old) (26). Four studies compared LVHIIT to no exercise, and one compared LVHIIT to a sham-exercise placebo. Four groups (10, 25–27) used cycling, and 1 group used jogging/running (28). There were no statistically significant differences in the patient baseline characteristics. All studies reported the exercise duration: 3 studies reported 12 weeks, 1 study for 1 weeks and 1 for 16 weeks. All studies included in our meta-analysis were published between 2016 and 2022, and the total sample size was 119. The detailed information on medication intake was shown in **Table 2**. The mean Jadad score ranged from 3 to 5. The main limitation of the included studies was the blinding methods. The Jadad scores for each study are also presented in **Table 1**.

**Table 1:** Characteristics of included studies.

No.	Author	Year	Low volume HIIT group					Control group					
			Number	Age (Years)	Male	Duration (Weeks)	Intervention	Number	Age (Years)	Male	Duration (Weeks)	Intervention	Jadad Score
1	Alvarez et al.	2016	13	46.0 ± 3.0	0	16	Jogging/running; 3 times/week; Progressive exercise program intervals interspersed with recovery periods of low-intensity walking; Reach 90–100% and less than 70% heart rate of their age predicted.	10	43.0 ± 2.4	0	16	Non-exercising	3
2	Winding et al.	2017	13	54.0 ± 6.0	7	11	Cycling; 3 times/week;20 min consisting of 1 minute at 95% Workload peak and 1 min 20% Work load peak recovery.	13	57 ± 7	5	11	Non-exercising	4
3	Afousi et al.	2018	18	54.78 ± 6.19	9	12	Cycling; 3 times/week; 1.5 min at 85–90% HRmax separated by 2 min at 55–60% HRmax.	17	54.24 ± 5.61	9	12	Non-exercising	3
4	Way et al.	2020	12	56.9 ± 2.1	7	12	Cycling; 3 times/week; 4 min high-intensity at 90% V̇_O2_peak and a 5 min cool-down at 50% V̇O2peak	11	51.9 ± 1.4	7	12	Sham-exercise placebo	4
5	Li et al.	2022	13	38.0 ± 6.0	13	12	Cycling; 5 times/week; 8 minutes 80%–95% HRmax and recovery at 25% intensity.	12	40 ± 7	12	12	Non-exercising	5

**Table 2:** Medication intake of patients.

Study	Medication intake (Control: LVHIIT), n
Alvarez et al.	Metformin 10:13; Glibenclamide 8:12; ACE inhibitor 3:3; Levothyroxine 1:1.
Winding et al.	Metformin 6:1; DPP-4 inhibitor 0:3; Sulfonylureas 1:3; GLP-1 analogues 1:2.
Afousi et al.	Diuretic 8:9; ACE inhibitors 5:4; Angiotensin blockers 4:3; Metformin10:9; Sulfonylureas 8:8; DPP-4 inhibitors 4:6; Statins 7:8.
Way et al.	Anti-Hyperglycemic 12:11; Anti-Hypertensive 8:6; Lipid Lowering 5:7.
Li et al.	Metformin 7:6; Sulfonylureas 3:3; DPP-4 inhibitors 2:3; Alpha-glucosidase inhibitor0:1.

### Primary outcomes

#### Fasting blood glucose

**Figure 2:**
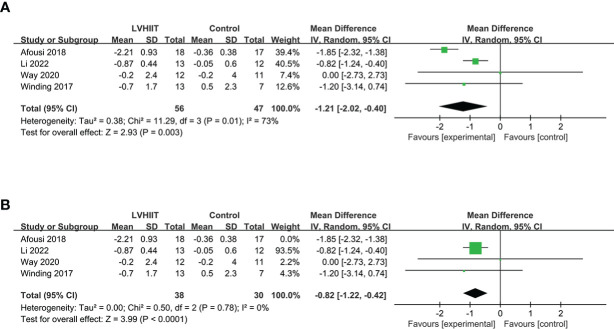
**(A)** Forest plot for the meta-analysis of **(A)** fasting blood glucose and **(B)** after sensitivity analysis.

Four studies examined pre- and post-LVHIIT FBG levels (10, 25, 27, 28). The results showed a significant difference in FBG in the LVHIIT group compared with the control group (RR= -1.21; 95% CI -2.02— -0.40, p = 0.0032), and there was significant heterogeneity (I^2^ = 73%, P = 0.01; **Figure 2A**). After removing the Winding et al. study (10), the heterogeneity became nonsignificant (I^2^ = 0%, P = 0.78), and the overall effect of exercise remained significant (RR= -0.82; CI -1.22 – -0.42, p = 0.0032; **Figure 2B**).

#### HbA1c

**Figure 3:**
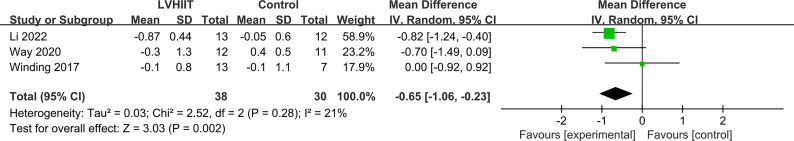
Forest plot for the meta-analysis of percentage of HbA1c.

Three studies examined changes in HbA1c levels (10, 25, 27). Our meta-analysis indicated that LVHIIT can significantly reduce the HbA1c levels (RR= -0.65; 95% CI= -1.06 – -0.23, p = 0.002; **Figure 3**), and there was nonsignificant heterogeneity (I^2 =^ 21%, p = 0.28).

#### HOMA-IR

**Figure 4:**
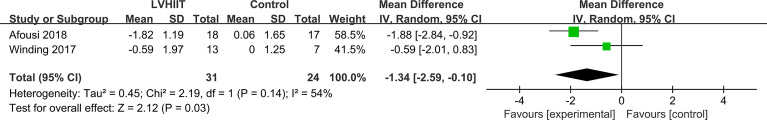
Forest plot for the meta-analysis of HOMA-IR.

Only two studies examined HOMA-IR levels (10, 28). As shown in **Figure 3**, HOMA-IR levels were significantly lower in the LVHIIT group than in the control group (RR= -1.34; 95% CI -2.59— -0.10, p = 0.03; **Figure 4**), and there was significant heterogeneity (I^2^ = 54%, P = 0.14).

### Secondary outcomes

#### Fasting insulin

**Figure 5:**
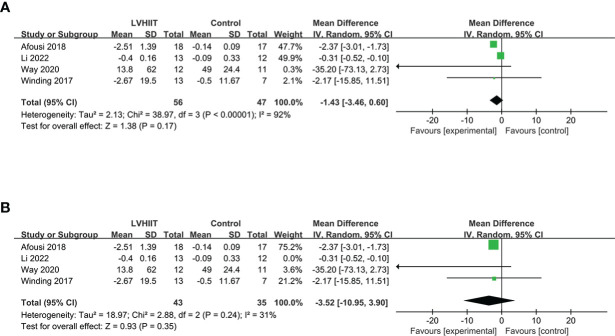
Forest plot for the meta-analysis of **(A)** fasting insulin and **(B)** after sensitivity analysis.

Four studies examined pre- and post-LVHIIT fasting insulin levels (10, 25, 27, 28). Our results revealed that LVHIIT did not significantly change the fasting insulin level compared to the control group (RR= -1.43; 95% CI = -3.46 – 0.60, p =0.17; **Figure 5A**), and there was significant heterogeneity (I^2 =^ 92%, p <0.00001). After removing the study by Li et al. (25), the overall effect of LVHIIT remained nonsignificant (RR= -3.52; 95% CI = -10.95 – 3.90, p =0.35; **Figure 5B**), and the level of heterogeneity was lower (I^2 =^ 31%, p =0.24).

#### BMI and body mass

**Figure 6:**
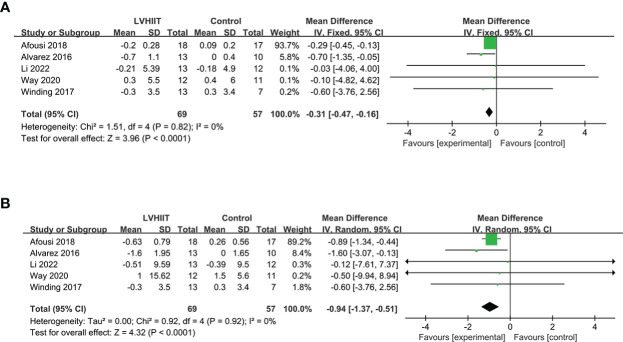
Forest plot for the meta-analysis of **(A)** body mass index and **(B)** body weight.

All five included studies examined BMI and body mass (10, 25–28). Our results revealed that LVHIIT reduces BMI (RR = -0.31, 95% CI = -0.47 – -0.16, p<0.0001; **Figure 6A**) and body mass (RR = -0.94, 95% CI = -1.37 – -0.51, p<0.0001; **Figure 6B**), and there was nonsignificant heterogeneity (I^2^ = 0%, P =0.82 and I^2^ = 0%, P =0.92, respectively).

#### Blood lipid metabolism

**Figure 7:**
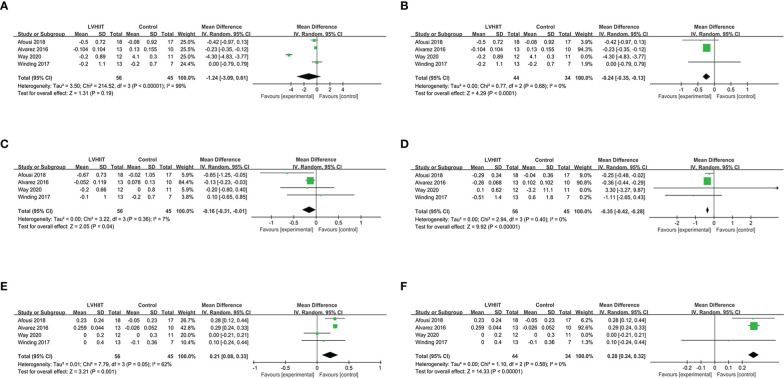
Forest plot for the meta-analysis of lipid profile **(A)** total cholesterol and **(B)** after sensitivity analysis; **(C)** low density lipoprotein; **(D)** triglyceride; **(E)** high density lipoprotein and **(F)** after sensitivity analysis.

Four studies (10, 26–28) examined data relating to blood lipid indicators. For total cholesterol, our meta-analysis indicated that there was no difference after a period of LVHIIT (RR = -1.24, 95% CI = -3.09—0.61, p=0.19; **Figure 7A**), and there was a significant level of heterogeneity (I^2^ = 99%, P <0.00001). However, after excluding one study, LVHIIT was found to significantly reduce total cholesterol (RR = -0.24, 95% CI = -0.35 to -0.13, p<0.0001; **Figure 7B**), and the heterogeneity became nonsignificant (I^2^ = 0%, P =0.68). For high-density lipoprotein, the meta-analysis showed that there was a significant difference between the LVHIIT group and the control group (RR = 0.21, 95% CI = 0.08 – 0.33, p=0.001; **Figure 7E**), and there was a significant level of heterogeneity (I^2^ = 62%, P=0.05). After removing the study by Way et al., the results still showed that LVHIIT increased plasma HDL levels (RR = 0.28, 95% CI =0.24—0.32, p<0.00001), but the heterogeneity was nonsignificant (I^2^ = 0%, P =0.58; **Figure 7F**). Overall, LVHIIT can reduce TC, LDL and triglyceride levels and increase HDL levels.

#### Cardiorespiratory fitness parameters

**Figure 8:**
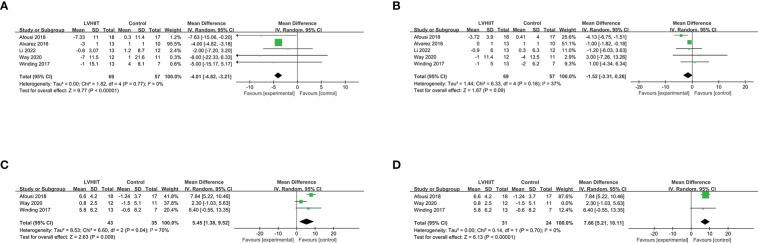
Forest plot for the meta-analysis of cardiorespiratory fitness **(A)** systolic blood pressure and **(B)** diastolic blood pressure; **(C)** relative VO2 peak; **(D)** after sensitivity analysis.

All five studies included the SBP and DBP (10, 25–28). Our results indicated that LVHIIT can reduce the SBP (RR =-4.01, 95% CI = -4.82 – -3.21, p<0.0001; **Figure 8A**) but not DPB (RR =-1.52, 95% CI = -3.31 – 0.26, p=0.09; **Figure 8B**) with nonsignificant heterogeneity (I^2^ = 0%, P =0.77 and I^2^ = 37%, P =0.18, respectively). Three studies reported the VO_2peak_ and our result showed that LVHIIT significantly improved VO_2peak_ compared to the control group (RR= 5.45; 95% CI = 1.38 – 9.52, p =0.009; **Figure 8C**) and there was significant heterogeneity (I^2 =^ 70%, p =0.04) (10, 26, 27). After removing the study by Way et al. (27), the overall effect of LVHIIT remained significant (RR= 7.66; 95% CI = 5.21 – 10.11, p<0.00001; **Figure 8D**), and the level of heterogeneity was lower (I^2 =^ 0%, p =0.70).

## Discussion

Increased evidence has shown that physical exercise is an essential component of all effective interventions for the treatment and prevention of T2DM. As different types of exercise bring different benefits to patients, a series of clinical trials and meta-analyses have been performed to determine the positive function of each type of exercise. Earlier studies have shown that aerobic exercise, resistance training and HIIT independently have beneficial effects on preventing T2DM (5). As T2DM patients often report a “lack of time” as one barrier to regular exercise (29), LVHIIT may be a more time-effective strategy. LVHIIT has already been proven to improve cardiovascular health in T2DM patients. However, the effect of LVHIIT on diabetes-related indicators such as glycaemic control, insulin level, and HbA1c remain unclear. To evaluate this type of exercise and obtain higher-level evidence, we performed this meta-analysis.

Hyperglycaemia is the key characteristic of diabetes mellitus and is the main cause of complications in the heart, vasculature, eyes, kidneys and nerve system (30). Almost all types of exercise can reduce hyperglycaemia by improving insulin resistance in peripheral organs, such as skeletal muscle, liver and adipocytes (31–34), which will enhance blood glucose uptake and transport. Jelleyman et al. reported in their meta-analysis that regular HIIT can significantly reduce fasting glucose in metabolic syndrome or T2DM but not in healthy people compared to no exercise patients (35). Our present research found a reduction in fasting glucose levels among T2DM patients after LVHIIT intervention. Little et al. reported that LVHIIT can reduce hyperglycaemia by enhancing insulin signaling, the insulin-stimulated glucose disposal rate, glucose transporter protein (GLUT4) levels, and mitochondrial capacity in muscle, which further confirms our results (34). HOMA-IR, a model for estimating insulin sensitivity through glucose concentrations and fasting insulin, was also improved in the LVHIIT group (36). The level of heterogeneity was higher for this outcome, which may be due to different blood sampling times and the specific calculation model they used. Thus, we can assume that LVHIIT can significantly improve hyperglycaemia and insulin resistance in T2DM patients. HbA1c is another indicator for blood glucose concentration and is a very important predictor for the incidence of complications and death related to diabetes. A previous study reported that each 1% increase in HbA1c is associated with a 37% increase in diabetic microvascular complications and a 21% increase in the risk of mortality. Thus, HbA1c is also a crucial marker for evaluating the therapeutic method of diabetes (37, 38). First, the formation of HbA1c is related to the average blood glucose concentration at three months. Second, a previous study showed that extent to which HbA1c levels decrease depends on the type and volume of exercise (39). Our results showed that medium- or long-term (11-16 weeks) LVHIIT can significantly reduce HbA1c and benefit T2DM patients. Notably, these glycaemic-related indicators were also improved in a short-term (2 weeks) experiment. Unfortunately, the study had a small sample size, and the evidence level of the study design was not high (34). Due to the improvement in insulin resistance, fasting insulin should be lower. However, our result shows a lower tendency of insulin without statistical significance. This result may partly explain why exercise improved insulin signaling in peripheral tissue rather than enhancing the insulin secretion function of β-cells (40). Ishiguro et al. assumed that insulin improvement may be restricted in patients with impaired basal insulin secretion with severe insulin resistance or impaired basal insulin secretion (41).

A previous meta-analysis that only included aerobic training and resistance training showed that BMI and body mass had nonsignificant reductions (42). Jelleyman et al. reported that HIIT can reduce body mass and BMI compared to the control group. However, the included study had relatively inconsistent baselines, as the included studies had different types of patients, such as healthy, overweight, T2DM and other chronic diseases (35). Our results further confirmed that LVHIIT can help reduce BMI and body weight in T2DM patients. However, body composition, such as body fat percentage, waist circumference and waist-hip ratio, should be further investigated. Our study also showed that LVHIIT significantly improved the blood lipid profile. The study by Way et al. contributed to the overall heterogeneity of this outcome because of the baseline characteristics of their patients (some patients were taking lipid-lowering medication). The main shortcoming for this outcome is the lack of the consistent dietary interventions across studies. Thus, future studies should provide a more consistent energy intake to determine the real efficiency of LVHIIT and plasma lipid metabolism. Corres et al. have reported that compared to high-volume moderate intensity continuous training, LVHIIT contributes to better improvements in cardiopulmonary function which is verified by our meta-analysis. Furthermore, they report that LVHIIT has a lowest withdraw rate compared to other type of exercises (43). Afousi et al. report that LVHIIT can decrease the oscillatory shear-induced improvement inflow-mediated dilatation and outward artery remodeling in T2DM patients compared to MICT (26).

Admittedly, there were some limitations in this meta-analysis. First, the number of participants in the studies was relatively small. Second, the LVHIIT period is approximately 11 to 16 weeks; therefore, it is difficult to determine the effects of shorter or longer LVHITT interventions. Third, there was always a certain amount of heterogeneity because there is no fully standardized LVHIIT protocol for T2DM patients. Fourth, liver dysfunction is tightly associated with T2DM (44), while the parameters of liver function were lacked in present studies. Lastly, the missing negative and unpublished data in the original studies may have led to publication bias and skewed our conclusions. Thus, we suggest that robust RCTs with large sample sizes and a standard protocol with more outcome parameters be performed in future studies to obtain more accurate data and verify our results.

## Conclusion

In conclusion, this systematic review demonstrates that LVHIIT is an effective intervention for improving the metabolism of T2DM patients. Our results indicate that LVHIIT can reduce fasting blood glucose, HbA1c, insulin resistance, and body mass. Moreover, LVHIIT can improve the blood lipid profile, SBP and relative VO_2peak_. Nevertheless. Because of the current limitations of the included studies, multicenter, large-scale, prospective RCTs with more stable baselines should be performed to validate the present results.

## Data availability statement

The original contributions presented in the study are included in the article/supplementary material. Further inquiries can be directed to the corresponding authors.

## Author contributions

ZW and XZ participated in the design of this study. YP and ZW drafted the manuscript, YP, YO, and KW collected and analysis the data, XZ critically revised the manuscript. All authors have read and approved the final manuscript.
